# Glycyrol Prevents the Progression of Psoriasis-like Skin Inflammation via Immunosuppressive and Anti-Inflammatory Actions

**DOI:** 10.3390/ijms242417335

**Published:** 2023-12-11

**Authors:** Yuanyuan Liu, Yanxia Fu, Ziwei Zhu, Shanzao Chen, Li Tong, Qun Wei

**Affiliations:** Beijing Key Laboratory of Genetic Engineering Drug and Biotechnology, Department of Biochemistry and Molecular Biology, College of Life Sciences, Beijing Normal University, Beijing 100875, China; liuyuanyuan@bj80.com (Y.L.); 20190055@ccmu.edu.cn (Y.F.); 201311201008@bnu.edu.cn (Z.Z.); cshanzao@163.com (S.C.)

**Keywords:** cyclosporine A, imiquimod, immunosuppression, inflammation, nephrotoxicity

## Abstract

Glycyrol (GC) is one natural active product. Imiquimod-induced psoriasis-like Balb/c mouse models were established. The model mice were intraperitoneally injected with cyclosporine A (CsA) and GC for 8 days followed by a series of biological detections. GC had little toxicity according to the levels of peripheral blood cells, hemoglobin, blood urea nitrogen (BUN), and serum creatinine (CRE), while CsA significantly increased the levels of BUN and CRE. GC decreased the splenic index and reduced the expressions of IL-6, IL-23, and CXCL-3 in the model mice and IL-6, CXCL-1, and CXCL-2 in the inflammatory HaCaT cells. The half inhibition concentration (IC_50_) of GC on HaCaT cells was 29.72 μmol/L, resulting in improved apoptosis, enhanced expressions of p21, BAX, and BIK, and reduced expressions of BCL-2. GC is an immunosuppressive agent against psoriasis-like symptoms by anti-inflammatory effects, which provides a strategy for the discovery of anti-psoriatic natural products.

## 1. Introduction

Psoriasis is a common chronic skin disorder with a tendency to relapse even if its symptoms are controlled. Currently, approximately 2–3% of individuals worldwide suffer from psoriasis [1]. The typical symptoms include skin scaling and erythema, obvious thickening of the skin, epidermal hyperplasia, and abnormal proliferation and differentiation of keratinocytes [2]. More importantly, it can seriously affect the mental health of patients [3]. Patients with moderate to severe psoriasis may need long-term or lifelong systemic pharmacotherapy. Unfortunately, long-term safe and effective anti-psoriasis drugs are currently not available.

Psoriasis is thought to be an immune system problem that causes skin cells to grow faster than usual, where infection-fighting cells attack healthy skin cells by mistake [4,5]. The typical symptoms of psoriasis are skin lesions with clear red-speckled plaques. Its histopathological features include hyperkeratosis caused by the abnormal proliferation of keratinocytes, parakeratosis caused by the retention of keratinocyte cell nuclei, acanthosis, and infiltration of inflammatory cells into the dermis [6]. The excessive proliferation and abnormal differentiation of keratinocytes are the major features of psoriasis, leading to most of the skin symptoms. Although the complex interaction between pro-inflammatory factors and keratinocytes plays an important role in the progression of psoriasis, much remains to be understood about its causes [7]. Clinically applied anti-psoriatic medications include methotrexate, acitretin, cyclosporine A (CsA), fumaric acid esters, apremilast, and dimethylfumarate [8]. Recently, other treatments, including traditional Chinese medicines [9,10], natural products such as curcumin [11], celastrol [6], capsaicin [12], luteolin [13], and photodynamic therapy [14], have received attention and are effective and safe.

Psoriasis is considered to result from an interplay of genetic, environmental, infectious, and lifestyle factors [14]. The main pathways involve the hypothalamic–pituitary–adrenal axis, sympathetic–adrenal–medullary axis, GPR43-mediated skin inflammation, peripheral nervous system, and immune system [15,16]. Based on the importance of IL-23/IL-17 axis, Bruton’s tyrosine kinase inhibitors can suppress imiquimod (IMQ)-induced psoriasis-like inflammation in mice through the regulation of IL-23/IL-17A in innate immune cells [17,18]. Moreover, the small molecular bromodomain and extraterminal domain (BET) inhibitors and the inhibitors of interleukin-2-inducible T-cell kinase (ITK) may become effective therapeutics for psoriasis based on different pathways [19,20].

It is very important to consider the potential adverse events and cumulative toxicity risks associated with the long-term use of medications for the treatment of psoriasis. CsA is a cyclic undecapeptide discovered in the 1970s to possess a potent inhibitory action on T lymphocytes. As a widely used immunosuppressant, CsA is an effective treatment for several immune-related diseases such as rheumatoid arthritis, multiple sclerosis, and psoriasis [1,21,22] and transplantation [23]. Up to now, it remains one of the most effective and rapidly acting treatments for psoriasis [24]. However, it has severe nephrotoxicity when long-term use and its oral dose is limited [8].

IMQ is a toll-like receptor (TLR7 and TLR8) agonist. It binds to TLR7 in epidermal plasmacytoid dendritic cells and macrophages and induces the secretion of IFN-α, IL-23, IL-17, and IL-22 [25]. Topically applied IMQ produces symptoms such as epidermal hyperkeratosis, parakeratosis, stratum spinosum hypertrophy, blood vessel formation, and inflammatory cell infiltration. Since these pathological features resemble psoriasis, the IMQ-induced psoriasis-like animal model is often used in psoriasis research [26,27].

Natural products are an important resource of active pharmacological ingredients [9,28]. Moreover, natural active ingredients are usually derived from traditional herbal medicines that have hundreds or thousands of years of history [29]. Glycyrol (GC), a coumarin compound, is extracted from *Glycyrrhiza uralensis* Fisch which is distributed worldwide, except for Antarctica [30]. *Glycyrrhiza uralensis* is used as a sweetening agent, a flavor in food and chewing gums, and as a depigmentation agent in cosmetics; more importantly, it is a common component of traditional Chinese medicines [31]. *Glycyrrhiza uralensis* has anti-hepatitis C virus, antibacterial, anti-inflammatory, anti-angiogenic, antioxidative, and anti-allergenic activities with little toxicity [28,32,33]. GC has wide pharmacological activities. It is believed that psoriasis is the result of a network of interactions between immune-associated cytokines and inflammatory factors. GC is known to have a pronounced anti-inflammatory effect [21,32]. GC has very low murine toxicity with an oral LD_50_ exceeding 2000 mg/kg [21]. However, the effects of GC as a major component of *Glycyrrhiza uralensis* have not been deeply explored. 

Here, we examined the effect of GC on the psoriasis-like mouse model and compared it with an established active drug, CsA, and we investigated its mechanisms of action in the mouse model and in a human keratinocyte cell line. Also, its toxicity was examined, especially in relation to nephrotoxicity. GC was found to be highly effective and has low toxicity.

## 2. Results

### 2.1. GC Alleviates Psoriatic Dermatitis

**Figure 1:**
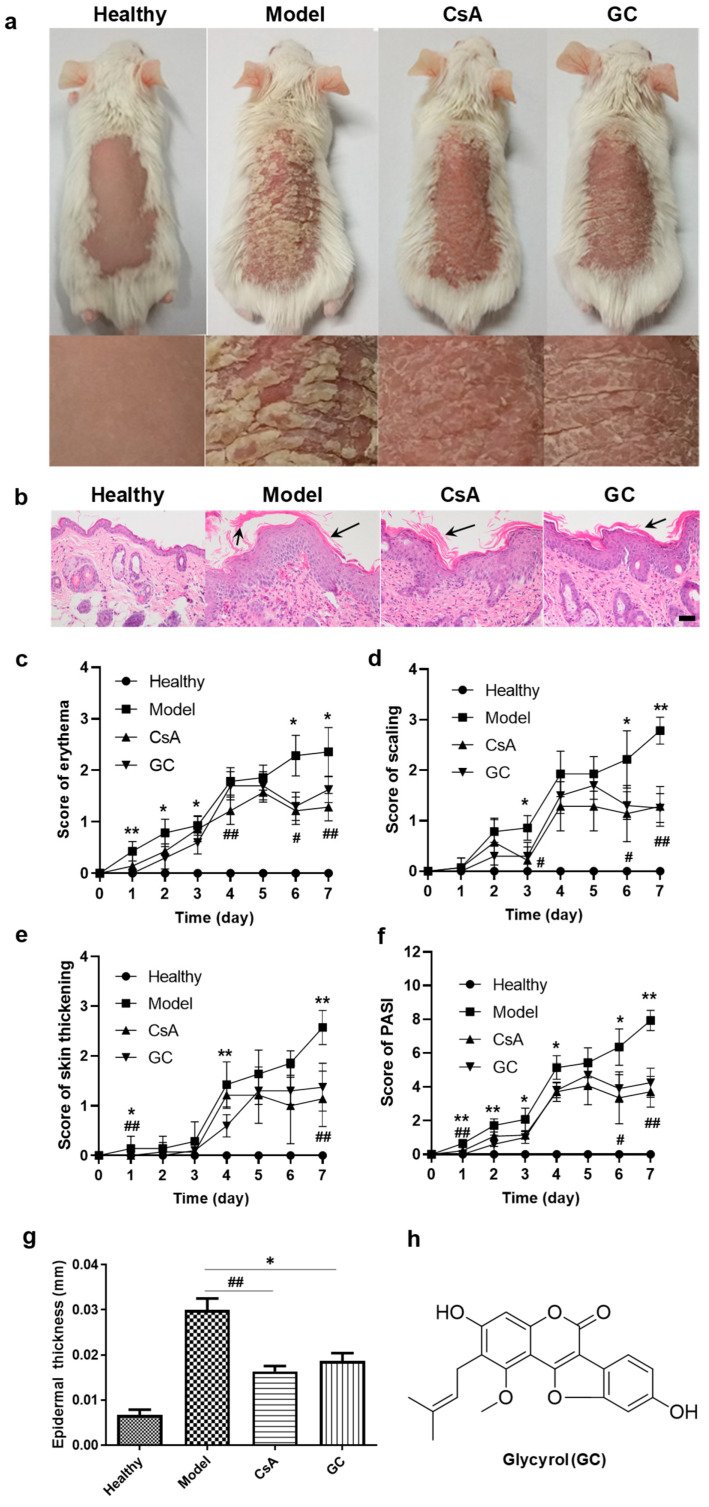
Appearance of the mice and skins and the related symptom levels on Day 7. (**a**) The appearance of the mice in the various groups. (**b**) The pathology images of the skins. The scores on erythema (**c**), scaling (**d**), skin thickening (**e**), and psoriasis area and severity index (PASI, (**f**)). The epidermal thickness (**g**). The structure of glycyrol (GC, (**h**)). Scale bar = 100 μm. *, *p* < 0.05, **, *p* < 0.01, GC vs. Model; #, *p* < 0.05, ##, *p* < 0.01, cyclosporine A (CsA) vs. Model; *n* = 6. The arrows indicate skin scaling.

Some studies have shown that skin inflammation in response to IMQ application in BALB/c mice develops more rapidly and causes more flaky and inflamed skin than in C57BL/6 mice [27]. In this study, the application of IMQ resulted in skin lesions on the mouse’s back on Day 1, marked scaling from Day 2, and significant skin thickening from Day 4. In contrast, GC’s treatment remarkably reduced the skin inflammation; so did CsA’s treatment (Figure 1). After the treatment for 5 days, the anti-psoriasis effects of CsA and GC were dramatic, with reduced scores for erythema, scaling, and skin thickening, as well as the psoriasis area and severity index (PASI) (Figure 1c–f). On Day 7, the model group still showed the typical symptoms of psoriatic dermatitis; whereas they were very weak in the other groups (Figure 1a,b). However, the mice in the CsA group appeared unhealthy with dry and dull fur (Figure 1a), indicating the possible toxicity of CsA [8]. All the untreated skin looked normal.

The pathological sections of psoriatic skin revealed the formation of plaques with significant desquamation, erythema, and thickening of the skin (Figure 1f). There was also severe acanthosis of the epidermis, significant epidermal hyperplasia, and the disappearance of granules. Micro-abscesses were formed, and thickening of the epidermis and infiltration of inflammatory cells into the dermis were observed (Figure 1f). Epidermal thickening was significantly reduced in the two treatment groups on Day 7 (Figure 1g). 

We conclude that both CsA and GC dramatically ameliorate psoriatic skin inflammation, with reduced epidermal thickening, a downward extension of the epidermal rete ridges, and little infiltration of mononuclear cells into the dermatitis, but that the long-term administration of CsA may be somewhat toxic.

### 2.2. GC Shows No Detectible Effects on the Functions of the Kidney and Liver

**Figure 2:**
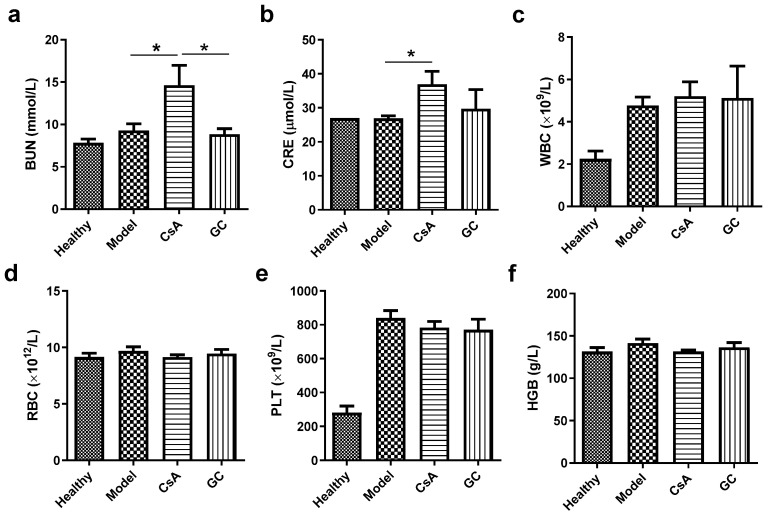
Profiles of the biochemical indicators and peripheral blood counts of the mice in the various groups on Day 7. The levels of blood urea nitrogen (BUN, (**a**)), serum creatinine (CRE, (**b**)), white blood cells (WBC, (**c**)), red blood cells (RBC, (**d**)), platelets (PLT, (**e**)), and hemoglobin (HGB, (**f**)) in the peripheral blood. *, *p* < 0.05; *n* = 6.

GC showed no detectable effects on the levels of blood urea nitrogen (BUN) and serum creatinine (CRE), which were the major indicators of nephrotoxicity. In contrast, CsA did increase the levels of BUN and CRE markedly (Figure 2a,b). In this study, both GC and CsA showed no effects on the major peripheral blood cell types, i.e., white blood cells (WBC), red blood cells (RBC), platelets (PLT), and hemoglobin (HGB) (Figure 2c,f). It is well known that nephrotoxicity may become a significant problem during the long-term administration of CsA [34]. While CG was relatively safe compared with CsA in this study.

### 2.3. GC Attenuates IMQ-Induced Immune Stimulation 

**Figure 3:**
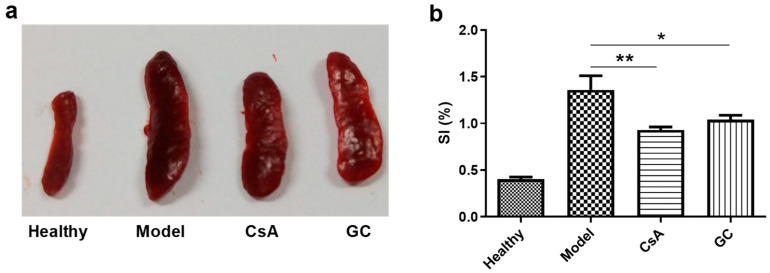
Appearance of the mouse spleens in the various groups (**a**) and the splenic indexes (SIs, (**b**)). *, *p* < 0.05; **, *p* < 0.01; *n* = 6.

The topical application of IMQ to the back skins of mice led to strong immune stimulation with the marked hypertrophy of the spleen in the model group (Figure 3a), and this effect was reduced, but not eliminated, in the two treatment groups (i.e., the CsA and GC groups). Moreover, the splenic indexes (SIs) of the CsA and GC groups were significantly lower than that of the model group, though higher than that of the healthy group (Figure 3b). All these findings indicate that GC and CsA have similar suppressive effects on IMQ-induced immune stimulation in psoriasis. 

### 2.4. GC Alleviates the Inflammatory Response of Lipopolysaccharide-Induced HaCaT Cells

**Figure 4:**
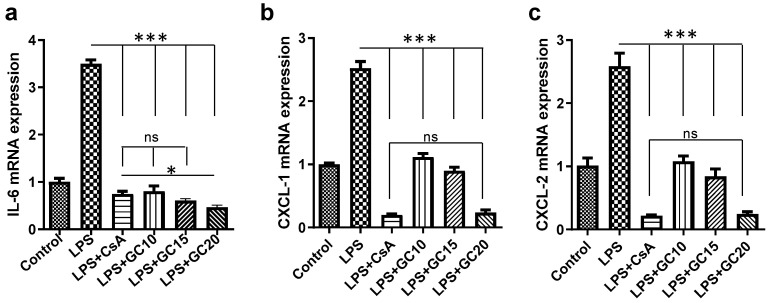
Cytokine transcriptional levels in the HaCaT cells. “Control” indicates untreated cells. “LPS” indicates lipopolysaccharide (LPS)-treated cells. “LPS + CsA” refers to the addition of 10 μmol/L cyclosporine A (CsA) to LPS-treated cells. “LPS + GC” indicates the addition of various concentrations of glycyrol (GC) to LPS-treated cells. Cells were incubated with LPS or LPS/treatments for 24 h before measurements were made. The mRNA expressions of IL-6 (**a**), CXCL-1 (**b**), and CXCL-2 (**c**). *, *p* < 0.05; ***, *p* < 0.001; ns, not significant; *n* = 3.

The levels of pro-inflammatory cytokines’ mRNAs (involving IL-6, CXCL-1, and CXCL-2) in the HaCaT cells increased strongly after lipopolysaccharide (LPS) exposure. However, the expressions of these pro-inflammatory cytokines were greatly reduced by CsA and GC (Figure 4). GC at 10 μmol/L significantly downregulated the mRNAs of CXCL-1 and CXCL-2. Moreover, the CXCL-1 and CXCL-2 suppressive effect of 20 μmol/L GC had no statistical difference with the effect of 10 μmol/L CsA (Figure 4b,c). The situation of IL-6 expressions was somewhat different from that of CXCL-1 and CXCL-2 expressions. GC at 10 and 15 μmol/L had similar suppressive effects to 10 μmol/L CsA, and the effect of 20 μmol/L GC was stronger than that of 10 μmol/L CsA (*p* < 0.05), indicating the strong anti-inflammatory effects of GC. 

**Figure 5:**
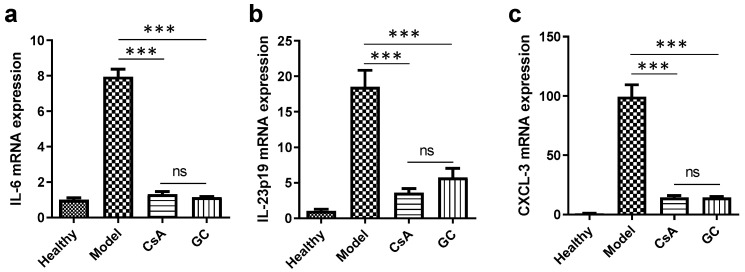
Transcriptional levels of the pro-inflammatory cytokines in the skins of the various groups. The mRNA expressions of IL-6 (**a**), IL-23p19 (**b**), and CXCL-3 (**c**). ***, *p* < 0.001; ns, not significant; *n* = 6.

The in vivo anti-inflammatory effects of GC and CsA were also examined. All the mRNA levels of the pro-inflammatory cytokines, including IL-6, IL-23p19, and CXCL-3, increased markedly in the model mouse skins (Figure 5). However, the levels were greatly reduced in the mouse skins treated with GC and CsA compared to those in the model mouse skins (*p* < 0.001). There was no significant difference between the GC and CsA groups. Therefore, the anti-inflammatory effect of GC is strong and comparable with that of CsA. 

### 2.5. GC Induces the Apoptosis of Keratinocytes

**Figure 6:**
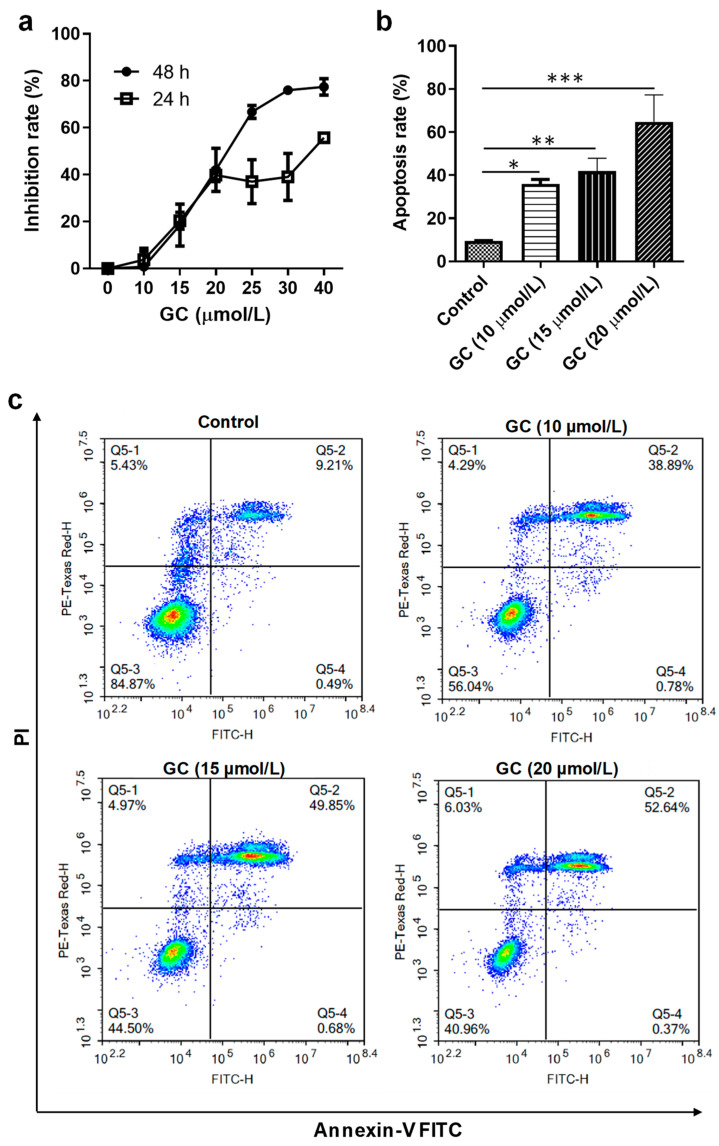
Effects of glycyrol (GC) on the growth of HaCaT cells. (**a**) The inhibition rates depending on GC concentrations. The rates of apoptosis (**b**) and the flow cytometry (**c**) of HaCaT cells after incubation with GC for 24 h. *, *p* < 0.05; **, *p* < 0.01; ***, *p* < 0.001; *n* = 3.

HaCaT cells are an immortal keratinocyte cell line derived from adult human skin and are often used in investigations on psoriasis [35]. GC showed a significant dose-dependent inhibition effect on the proliferation of HaCaT cells (Figure 6a). About an 80% inhibition rate of HaCaT cell proliferation was achieved when GC at 40 μmol/L was applied for 48 h. The HaCaT cell growth IC_50_ of GC was 29.72 μmol/L at 24 h and 16.20 μmol/L at 48 h, respectively. In our previous study [33], a similar result was received.

**Figure 7:**
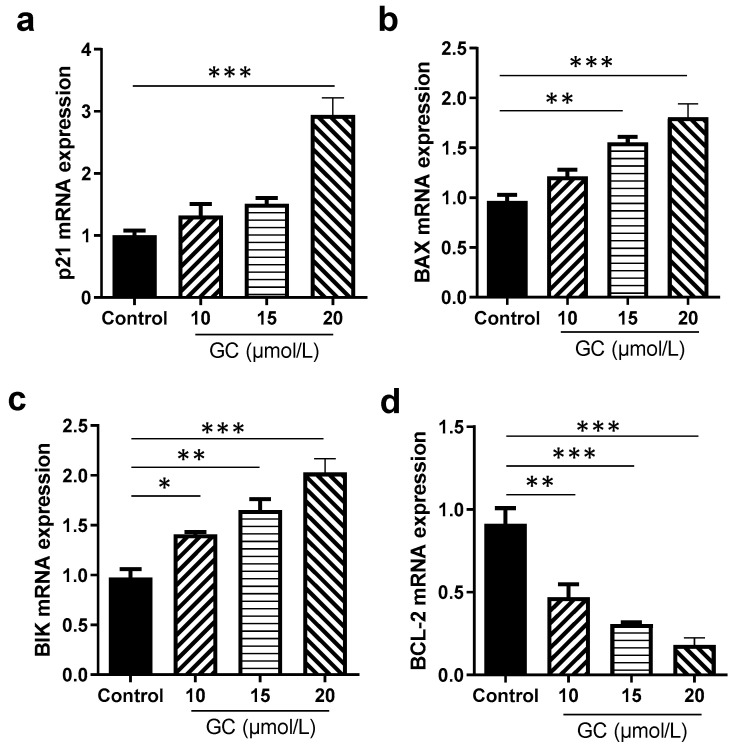
Effects of glycyrol (GC) on the expression of apoptosis-related genes in the HaCaT cells. (**a**) p21; (**b**) BAX; (**c**) BIK; and (**d**) BCL-2. *, *p* < 0.05; **, *p* < 0.01; ***, *p* < 0.001; *n* = 3.

The inhibitory mechanism of GC on the proliferation of HaCaT cells was investigated using flow cytometry. GC induced the remarkable apoptosis of the cells. Moreover, the promoting keratinocyte apoptosis effects of GC were of concentration dependence from 10 μmol/L to 20 μmol/L (Figure 6b). At 20 μmol/L, a more than 60% apoptosis rate was achieved. In the flow cytometry graph (Figure 6c), Q5-2 indicates the percentage of late apoptotic cells, Q5-4 shows the percentage of early apoptotic cells, Q5-3 represents the percentage of normal cells, and Q5-1 represents the percentage of primary necrotic cells. During the advanced phases of apoptosis, the integrity of cell membranes is impaired, facilitating the ingress of PI into cells and its subsequent binding to DNA, resulting in conspicuous red staining [36]. According to the flow cytometric results (Figure 6c), GC induced a strong apoptosis effect on the HaCaT cells. We further examined the effect of GC at various concentrations on the expressions of apoptosis-related genes. GC remarkably stimulated the expressions of pro-apoptosis genes, involving p21, BAX, and BIK, and inhibited the expression of an anti-apoptosis gene, BCL-2, and the effects were concentration-dependent (Figure 7). Therefore, the pro-apoptotic action of GC on keratinocytes is remarkable.

## 3. Discussion 

In this study, we confirmed the non-nephrotoxicity of GC on the psoriasis-like mouse model. It has been reported that the long-term administration of GC seems less toxic compared to CsA [21]. GC has also been shown to reduce the in vitro and in vivo combined toxicity of fumonisin B1 and cadmium by inhibiting the IRE1α-JNK axis [11].

Here, the transcriptional levels of IL-6, IL-23p19, and CXCL-3 were elevated in the psoriasis-like mouse model, and this effect was reduced more in the GC group. Since these cytokines are important effectors of the JAK/STAT and NF-κB signal transduction pathways, it is likely that GC regulates the differentiation of immune cells in the psoriasis-like mouse model, at least in part via these two pathways [37,38]. In a recently published article, the authors indicate that oestrogen has a dual potential in the pathogenesis of psoriasis, including suppression of inflammation by enhancing IL-10 production and the enhancement of inflammation by induction of IL-22 and IL-23 expression [39]. IL-23 plays a prominent role in the development of psoriasis [40]. Moreover, some natural active products, such as D-chiro-inositol and glycerol, show significant anti-psoriasis activities [41,42].

A substantial release of pro-inflammatory cytokines by Th1 and Th17 cells is the major cause of keratinocyte proliferation, i.e., inflammatory cell infiltration is a distinctive feature of psoriasis [34]. GC may have strong anti-inflammatory and antioxidant actions [43,44]. One of the remarkable features of psoriasis is the proliferation of keratinocytes. In this study, we demonstrated that GC inhibited the proliferation of HaCaT cells and induced their death. LPS is usually used for stimulating HaCaT cells to make an in vitro inflammation model [45,46,47]. LPS promotes the phosphorylation of JAK1 and STAT3, leading to increased cellular inflammation [45]. These findings suggest that GC inhibits immune cell activation by affecting immune-related factors and reducing the production of downstream inflammatory agents. 

In this study, the HaCaT cell line was used to test the apoptosis of keratinocytes induced by CG. The HaCaT cell line is a spontaneously immortalized, human keratinocyte line which has been widely used for studies of skin biology and differentiation [48]. It was found that HaCaT cells had a growth-differentiation difference depending on the surroundings compared with normal human keratinocytes (NHK) [49]. HaCaT cells are widely applied in psoriasis studies where the proliferation of HaCaT cells was definitely inhibited by the effective anti-psoriasis agents. The agents had a different half-maximal inhibitory concentrations (IC_50_) on the proliferation of HaCaT cells, such as 5 μM for 2-methoxyestradiol [35], 0.18 μM for flonoltinib maleate, 7.4 μM for anthralin [50], and 37.5 μg/mL for the ethanol extract of *Artemisia capillaris* [51]. In our study, the IC_50_ of GC on HaCaT cells was 29.72 μM at 24 h and 16.20 μM at 48 h. Therefore, an effective anti-psoriasis agent needs to maintain an appropriate inhibition concentration for the proliferation of HaCaT cells. Moreover, in the apoptosis experiment, the concentrations of GC were limited to relatively low values, i.e., less than 20 μM.

The pharmacological mechanisms of GC are based on its molecular structure. GC is a polyphenolic compound (Figure 1h). Polyphenolic compounds, such as curcumin, have wide pharmacological actions, including anti-inflammatory and immunomodulatory activities, and one important mechanism is their reaction oxygen species (ROS) scavenging function [52]. The anti-psoriasis effect of curcumin is also reported [53], and the regulation of Th-17-related inflammatory factors is considered as one of the mechanisms [54]. Moreover, the relationship between the anti-psoriasis effect of GC and the molecular structure of GC needs deep exploration. The expressions of GC and/or its metabolites in the skin of mice treated with IMQ also need detection.

## 4. Materials and Methods

### 4.1. Reagents

GC (C_21_H_18_O_6_, MW, 366.4, more than 95% purity, Figure 1h) was isolated and purified from *Glycyrrhiza uralensis* in our laboratory [33]. *Glycyrrhiza uralensis* was purchased from Tongrentang Chinese Medicine Pharmaceutical Group (Beijing, China), and authenticated by Jing Luo of the Department of Biochemistry and Molecular Biology, Beijing Normal University, where a voucher specimen (LJ-TRTGU-06) has been deposited. CsA (C_62_H_111_N_11_O_12_, MW, 1202.6) was purchased from TCI (Shanghai, China) Development Co., Ltd., Shanghai, China. IMQ Aldara^®^ 5% cream was purchased from 3M Health Care Limited (St. Paul, MN, USA). Cremophor EL, a polyoxyethylene castor oil derivative and nonionic surfactant, was obtained from Macklin Inc., Shanghai, China. AG RNAex Pro Reagent was obtained from Axygen, Corning, NY, USA. All other reagents were of analytical grade. Water manufactured with the Milli-Q^®^ system (Milli Q Reference, Darmstadt, Germany) was used throughout. 

### 4.2. Culture and Treatment of Cells

A HaCaT human keratinocyte cell line (1101HUM-PUMC000373, the National Infrastructure of Cell Line Resources of China) was obtained from the Cell Resource Center, Peking Union Medical College, the headquarters of the National Infrastructure of Cell Line Resource, Beijing, China. The cells were cultured in the minimum Eagle’s medium (MEM, Thermo Scientific, Waltham, MA, USA), supplemented with 10% fetal bovine serum, and incubated at 37 °C in a humidified atmosphere with 5% CO_2_. The medium was replaced every two days. For the cytotoxicity assay, HaCaT cells were inoculated in 96-well plates (5 × 10^3^ cells/well). After 12 h, they were treated with a series of concentrations of GC at 10, 15, 20, 25, 30, and 40 μmol/L, where the initial solvent dimethyl sulfoxide (DMSO) was less than 0.1% in the final culture media. After 24 or 48 h, CCK-8 (20 μL) was added to each well, and incubation continued for 4 h. Absorbance (optical density, OD) was measured with a microplate reader (POLARstar Omega, BMG Labtech, Ortenberg, Germany) at 450 nm, and inhibition rates were calculated as (OD_control_ − OD_GC_)/OD_control_ × 100%.

### 4.3. Animals

Balb/c mice are one of the commonly used psoriasis-like animal models, and female Balb/c mice are commonly used [13,26]. Female Balb/c mice (6–8 weeks old) were purchased from Beijing Vital River Experimental Animal Technology Co., Ltd., Beijing, China. The mice were housed under constant conditions of humidity (50 ± 5%) and temperature (25 ± 1 °C) with 12–12 h light–dark cycles. Food and water were available ad libitum. All animal procedures were approved by the Animal Ethics Committee of Beijing Normal University (CLS-EAW-2021-011) and were conducted in accordance with institutional guidelines.

### 4.4. Establishment and Treatment of the Psoriasis-like Mouse Model

GC and CsA were dissolved in the vehicle (5% DMSO/4% Cremophor EL/6% ethanol/85% saline), respectively. Twenty-four mice were randomly divided equally into four groups with six mice in each group, including the healthy, model, CsA, and GC groups. They were anesthetized using intraperitoneal (i.p.) injection of pentobarbital sodium with a dose of 50 mg/kg. Their back fur was partially shaved with an electric clipper, followed by topical administration of the depilatory cream (Veet Hair Removal Cream, Reckitt Benckiser (Gurgaon, India) Ltd.). For the mice in the healthy group, a generic petroleum jelly was spread on the exposed skin and the mice were i.p. injected with the vehicle (200 μL) every day for 8 consecutive days. For the model group, IMQ cream (62.5 mg) was spread on the bare backs of the mice which were i.p. injected with the vehicle (200 μL) every day from Day 0 to Day 7. The mice in the GC and CsA groups received IMQ, same as the mice in the model group, followed by i.p. injection with 200 μL of GC and CsA, respectively, at the dose of 20 mg/kg per mouse from Day 0 to Day 7. All the mice were killed on Day 7. The back skins were excised, and blood was collected for subsequent measurements.

### 4.5. Evaluation of the Severity of Psoriasis 

A modified scoring system, referred to as the clinical PASI, was used to evaluate the inflammatory state of the mouse dorsal skin [55]. The PASI measures three parameters: erythema, scaling, and thickening, each on a scale of 0–4: 0 (none; 1, slight; 2, moderate; 3, marked; and 4 very marked) giving a cumulative score of 0 to 12. Scoring was performed every day. One investigator did the scoring and was blinded to the details of the group, while another recorded the scores.

### 4.6. Evaluation of the Splenic Index 

The spleen is the major organ of the immune system, and an increased SI (spleen weight/body weight) is thought to reflect immune activation [56,57]. In this study, the spleens of mice were excised and weighed at the end of the experiment, and the SI was calculated.

### 4.7. Pathology

The excised dorsal skins of mice were fixed in 4% paraformaldehyde for 48 h and embedded in paraffin. Microtome sections of 4 μm thickness were prepared, deparaffinized, rehydrated, and stained with hematoxylin and eosin (H&E). A microscope equipped with a computer-controlled digital camera (Imager M1, Zeiss, Jena, Germany) was used to examine the sections. 

### 4.8. Peripheral Whole Blood Count Test

The toxicity of GC and CsA was investigated by measuring WBC, RBC, PLT, HGB, BUN, and CRE at the end of treatment, i.e., on Day 7. Peripheral whole blood was collected from the eye sockets of the mice and divided into two parts. One part was used for collecting serums and performing the analyses using a blood cell counter (SysMex XN-350, Kobe, Japan). The other part was placed in a heparinized tube for analysis using a hematologic analyzer (AU680, Beckman, Brea, CA, USA). 

### 4.9. Quantitative Real-Time PCR

Total RNA was isolated using Trizol reagents (BioTeke Corporation, Beijing, China) and was then reverse-transcribed with a Prime Script RT Reagent Kit (TaKaRa Bio, Kusatsu, Shiga, Japan). The conditions were as follows: 37 °C for 15 min and 85 °C for 5 s. Glyceraldehyde-3-phosphate dehydrogenase served as the reference in vivo and β-actin in vitro. Relative quantification was performed by the ΔΔCT method using the formula 2^−ΔΔCT^. The quantitative real-time PCR process consisted of initial denaturation for 5 min at 95 °C, 40 cycles at 95 °C for 5 s, and 60 °C for 34 s. The mRNA levels of IL-6, IL-23p19, and CXCL-3 in the mouse skin, and CXCL-1, CXCL-2, and IL-6 in HaCaT cells were analyzed using an ABI-sequence detection system (Applied Biosystems, Waltham, MA, USA). 

In the experiments, HaCaT cells were incubated with LPS for 24 h to simulate the environment of keratinocytes in psoriasis. Then, the HaCaT cells were incubated with CsA (10 μmol/L) or GC (10, 15, and 20 μmol/L, respectively) as the LPS + CsA group, the LPS + GC10 group, the LPS + GC15 group, and the LPS + GC20 group, respectively. In the in vivo experiment, the skins of mice of the various groups were excised and homogenized with 1 mL of AG RNAex Pro Reagent using a high-speed tissue homogenizer, followed by centrifugation at 12,000× *g* at 4 °C for 15 min. The total mRNA was extracted from supernatants.

The primer sequences used to detect inflammation-related gene expression in HaCaT cells are listed in Table 1, and those in the mice are listed in Table 2. 

### 4.10. Apoptosis Assay of HaCaT Cells 

The HaCaT cells were washed twice with phosphate-buffered solutions (PBS), stained according to the manufacturer’s instructions, and analyzed using a flow cytometer (BD Influx, BD Biosciences, San Jose, CA, USA). The frequencies of apoptotic and necrotic cells were analyzed with an Annexin V-FITC Apoptosis Detection Kit (Dojindo Molecular Technologies, Inc., Kumamoto, Japan). This method can differentiate viable cells (FITC negative, propidium iodide (PI) negative), early apoptotic cells with intact cell membranes (FITC positive), late apoptotic and secondary necrotic cells (FITC positive, PI positive), and primary necrotic cells (FITC negative, PI positive). The primer sequences designed to detect apoptosis-related gene expression in the HaCaT cells are listed in Table 3.

### 4.11. Statistical Analysis

Data are presented as mean ± standard derivation (SD). Differences between groups were analyzed by one-way analysis of variance (ANOVA) with the least significant difference (LSD) test in the post hoc test. All statistical analyses were performed using SPSS software (16.0, International Business Machines Corporation, Armonk, NJ, USA), and *p* < 0.05, *p* < 0.01, and *p* < 0.001 are considered to indicate significant differences, marked differences, and very marked differences, respectively.

## 5. Conclusions

In summary, GC had a strong anti-psoriasis effect by virtue of its immunosuppressive and anti-inflammatory activities, and its action strength is similar to CsA. More importantly, it has no effect on renal function, i.e., no nephrotoxicity, compared to the traditional immunosuppressive agent CsA which has significant nephrotoxicity. This study indicates that natural active ingredients may have advantages, including high effectiveness and low side effects, to resist immune diseases. We believe that GC, as a common natural product, holds promise as a low toxicity and effective medication for preventing the progression of psoriasis.

## Figures and Tables

**Table 1:** Primers for qPCR of human genes for detecting inflammatory factor mRNAs in HaCaT cells.

Primer	Base Sequence (5**′** to 3**′**)
β-actin (Forward primer)	AGGGAAATCGTGCGTGACAT
β-actin (Reverse primer)	TCCTGCTTGCTGATCCACAT
CXCL-1 (Forward primer)	CCCCAAGA ACATCCAAAGTG
CXCL-1 (Reverse primer)	GATGCAGGATTGAGGCAAG
CXCL-2 (Forward primer)	CCCATGGTTAAGAAAATCATCG
CXCL-2 (Reverse primer)	CTTCAGGAACAGCCACCAAT
IL-6 (Forward primer)	AGAGTAGTGAGGAACAAGCC
IL-6 (Reverse primer)	TACATTTGCCGAAGAGCCCT

**Table 2:** Primers for qPCR of mouse genes for detecting inflammatory factor mRNAs in mouse skin.

Primer	Base Sequence (5**′** to 3**′**)
GAPDH (Forward primer)	TGTGTCCGTCGTGGATCTGA
GAPDH (Reverse primer)	TTGCTGTTGAAGTCGCAGGAG
IL-6 (Forward primer)	CAACGATGATGCACTTGCAGA
IL-6 (Reverse primer)	CTCCAGGTAGCTATGGTACTCCAGA
IL-23p19 (Forward primer)	CTTTGAAGATGTCAGAGTCAAGCAG
IL-23p19 (Reverse primer)	ACATGCACCAGCGGGACATA
CXCL-3 (Forward primer)	CCCCAGGCTTCAGATAATCA
CXCL-3 (Reverse primer)	TCTGATTTAGAATGCAGGTCCTT

**Table 3:** Primers for qPCR of human genes for detecting apoptosis-related mRNAs in HaCaT cells.

Primer	Base Sequence (5**′** to 3**′**)
β-actin (Forward primer)	AGGGAAATCGTGCGTGACAT
β-actin (Reverse primer)	TCCTGCTTGCTGATCCACAT
p21 (Forward primer)	CGCTGCAGGACACATGGGGAGCCGAGCAGCG
p21 (Reverse primer)	TCGGCTCCCCATGTGTCCT
BAX (Forward primer)	CCAAGAAGCTGAGCGAGTGTC
BAX (Reverse primer)	TGAGGACTCCAGCACAAAGA
BIK (Forward primer)	TAGGATCCATGTCTGAAGTAAGACCCCTC
BIK (Reverse primer)	ACTCTCGAGTCACTTGAGCAGCAGGTG
BCL-2 (Forward primer)	CTGGGGGAGGATTGTGGCTTCT
BCL-2 (Reverse primer)	CTCCAACCCCCGCATCTCGGAC

## Data Availability

The data from this study can be obtained upon reasonable request from the corresponding author.
